# Improvement of pyrazolo[3,4-*d*]pyrimidines pharmacokinetic properties: nanosystem approaches for drug delivery

**DOI:** 10.1038/srep21509

**Published:** 2016-02-22

**Authors:** Giulia Vignaroli, Pierpaolo Calandro, Claudio Zamperini, Federica Coniglio, Giulia Iovenitti, Matteo Tavanti, David Colecchia, Elena Dreassi, Massimo Valoti, Silvia Schenone, Mario Chiariello, Maurizio Botta

**Affiliations:** 1 Dipartimento di Biotecnologie, Chimica e Farmacia, Università degli Studi di Siena, via Aldo Moro 2, 53100, Siena, Italy; 2 Lead Discovery Siena S.r.l., via Vittorio Alfieri 31, 53019, Castelnuovo Berardenga, Siena, Italy; 3 Consiglio Nazionale delle Ricerche, Istituto di Fisiologia Clinica and Istituto Toscano Tumori, Core Research Laboratory, Via Fiorentina 1, 53100, Siena, Italy; 4 Dipartimento di Scienze della Vita, Università degli Studi di Siena, via Aldo Moro 2, 53100, Siena, Italy; 5 Dipartimento di Farmacia, Università di Genova, Viale Benedetto VX 3, 16132, Genova, Italy; 6 Biotechnology College of Science and Technology, Temple University, Biolife Science Building, Suite 333, 1900 N 12th Street, Philadelphia, Pennsylvania 19122, USA.

## Abstract

Pyrazolo[3,4-*d*]pyrimidines are a class of compounds with a good activity against several cancer cell lines. Despite the promising anticancer activity, these molecules showed a poor aqueous solubility. This issue could threat the future development of pyrazolo[3,4-*d*]pyrimidines as clinical drug candidates. With the aim of improving their solubility profile and consequently their pharmacokinetic properties, we have chosen four compounds (1–4) on the base of their anti-neuroblastoma activity and we have developed albumin nanoparticles and liposomes for the selected candidates. Albumin nanoparticles and liposomes were prepared and characterized regarding size and ζ-potential distribution, polidispersity index, entrapment efficiency and activity against SH-SY5Y human neuroblastoma cell line. The most promising nanosystem, namely LP-2, was chosen to perform further studies: confocal microscopy, stability and drug release in physiological conditions, and biodistribution. Altogether, the obtained data strongly indicate that the encapsulation of pyrazolo[3,4-*d*]pyrimidines in liposomes represent an effective method to overcome the poor water solubility.

Neuroblastoma (NB) is the most common extracranial solid tumor in early childhood (the median age at diagnosis is 17 months). This tumor arises in the sympathetic nervous system as a result of genetic alterations occurring in neural crest cells^1^. Although NB is characterized by clinical and biological heterogeneity, on the base of age, tumor stage and genomic rearrangements (MYCN amplification and aneuploidy), the International Neuroblastoma Risk Group Staging System (INRGSS) classification divides patients in four groups, ranging from very low- to high-risk^2^. Up to 50% of NB patients have very aggressive tumors, associated with high risk of relapse and very poor prognosis with a 5-year event-free survival rate around 40%. Treatment strategies for high-risk patients are still far from satisfaction, especially considering the severe side effects of the most used drugs (i.e. cisplatin, etoposide, vincristine, doxorubicin and cyclophosphamide)^1^. Consequently, the search for novel drugs to improve NB treatment options still represents an outstanding pharmaceutical issue^3^.

In this context, Tyrosine Kinases (TKs) represent an interesting target for cancer treatment because of their involvement in several altered cellular pathways^4^. Molecular targeted therapies with Tyrosine Kinase Inhibitors (TKIs) are designed to disrupt signalling pathways responsible for the abnormal proliferation of cancer cells. Given the importance of this mechanism, several TKIs are currently under preclinical or clinical development^5^. A subclass of TKIs is represented by inhibitors of c-Src kinase, which is involved in cell proliferation, migration, invasion and angiogenesis as well as drug resistance development^6^,^7^. Since c-Src kinase has a strong connection with cancer development, several classes of small molecules (i.e. purines^8^, anilinoquinazolines^9^, quinolinecarbonitriles^10^, benzotriazines^11^, and thiazole-carboxamides^12^) have been developed to target this enzyme. Some outstanding c-Src inhibitors are: (I) Dasatinib, a dual Src/Abl TKI, FDA approved for chronic myeloid leukemia (CML) treatment, (II) Saracatinib^13^, currently in phase II clinical trials for the treatment of solid tumors, such as melanoma, prostate and gastric cancer, (III) Bosutinib^14^, a dual Src/Abl TKI, FDA approved for CML treatment^15^. Several studies confirmed that hyperactivated c-Src plays a key role in NB cell differentiation, adhesion and survival^16^,^17^,^18^,^19^,^20^. This TK was also associated to the progression of aggressive NB forms^21^,^22^. As a proof of concept of the effectiveness of inhibiting c-Src for NB treatment, recent studies demonstrated that the well-known c-Src inhibitor PP2 was able to inhibit cellular growth and to induce aggregation in NB cell lines^23^. Additionally the dual Src/Abl inhibitor Dasatinib was proved to be effective in reducing NB tumor growth *in vitro* and *in vivo*^24^.

The development of pyrazolo[3,4-*d*]pyrimidines as potential anticancer drugs, represents a major research focus of our group. This family of compounds showed a good cytotoxicity profile against several cancer cell lines: NB^25^,^26^,^27^, CML^28^, glioblastoma (GB)^29^, rhabdomyosarcoma (RMS)^30^, osteosarcoma (OS)^31^, prostate cancer (PC)^32^. The activity of pyrazolo[3,4-*d*]pyrimidines has been related to the inhibition of TKs such as Abl and Src-family proteins^33^. Recently, remarkable results have been obtained in several mouse models of cancer^25^,^28^,^30^. For instance, a small library of compounds with increased activity towards SH-SY5Y NB cell line was synthesized. The synthesis was driven by molecular modelling studies based on the X-ray crystal structure of c-Src in complex with one of our pyrazolo[3,4-*d*]pyrimidines. The selected lead compound was able to induce a tumor volume reduction greater than 50% in a NB subcutaneous xenograft mouse model^25^.

Given their unique physicochemical features, albumin nanoparticles and liposomes have showed a great potential as drug-carriers able to modify pharmacokinetic and pharmacodynamic properties of compounds. Encapsulation of a drug might result in an improved biodistribution, a higher stability, a controlled release, an ability to target sites otherwise not accessible, a decreased toxicity, and an enhanced bioavailability^34^. Oligomers built with albumin are widely used as nanoparticles for drug delivery^35^. This small protein, the most abundant in human plasma, presents very interesting features (i.e. biocompatibility, half-life of 19 days, water solubility and ability to bind a variety of different entities) and accumulates in malignant and inflamed tissues^36^. A breakthrough for albumin-based nanotechnology was the commercialization of Abraxane^©^, a solvent-free formulation of paclitaxel, where albumin binds paclitaxel to carry it through the endothelial cells to the tumor area^37^.

Liposomes are small vesicles characterized by a hydrophilic core surrounded by a phospholipid bilayer membrane whose composition can be adjusted to increase the therapeutic index of the encapsulated drug while minimizing its side effects. Liposomes are biodegradable and the Mononuclear Phagocyte System (MPS) uptake is strongly reduced when polyethylene glycol (PEG) is included in the membrane composition (Stealth Liposomes). Moreover, liposomes can be selectively trapped by the tumor due to enhanced permeation and retention^38^. Doxil, a PEGylated liposome-encapsulated form of doxorubicin, was the first FDA-approved nano-drug. It ensures prolonged blood circulation and lower cardiotoxicity when compared to free doxorubicin^39^.

Although the promising anticancer activity, pyrazolo[3,4-*d*]pyrimidines are usually characterized by a low water solubility profile^40^,^41^. Consequently, in this study we have further developed four pyrazolo[3,4-*d*]pyrimidines (1–4, Fig. 1) previously characterized for their activity against NB by enzymatic, cellular and *in vivo* assays (Table 1)^25^,^26^,^27^. Several strategies were applied to overcome this issue in recent years (i.e. introduction of hydrophilic moieties in solvent-exposed positions^42^, synthesis of prodrugs^43^ and inclusion in cyclodextrins)^40^,^41^.

With the aim of increasing the low water solubility of this class of compounds, in this study we have explored albumin nanoparticles and stealth liposomes as possible nanotechnologies.

## Results

### Pyrazolo[3,4-*d*]pyrimidines 1–4

**Figure 1:**
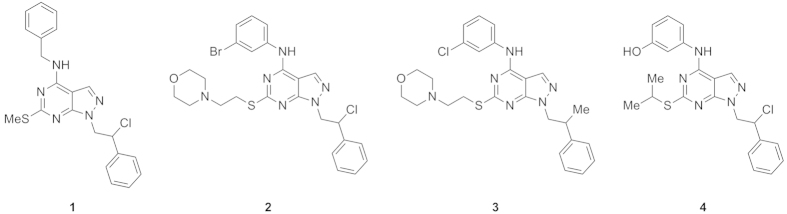
Structures of pyrazolo[3,4-*d*]pyrimidines 1–4.

The 4-amino substituted pyrazolo[3,4-*d*]pyrimidine ring represents a very interesting scaffold for the synthesis of molecules with antitumor activity. This structure is an isostere of ATP, the natural phosphorylating agent that binds TK. In our series of derivatives, the C4 amino function, essential for the interaction with the ATP-binding site, is attached to a *m*-substituted phenyl ring in derivatives 2–4 and a benzyl ring in derivative 1. Among the compounds previously characterized, pyrazolo[3,4-*d*]pyrimidine 1–4 were chosen for their ability to inhibit c-Src (with K_*i*_ values in the submicromolar range) and for their activity against NB cells^25^,^26^,^27^. Chemical structures of compounds 1–4 are shown in Fig. 1. *In vitro* ADME properties were also taken into account. In fact, the selected compounds demonstrated a very good metabolic stability (greater than 93% after incubation with human liver microsomes). Unfortunately, this favourable property was also associated with a low aqueous solubility and a medium-low passive permeability through membrane. Enzymatic assays and ADME properties of compounds 1–4, as previously described^25^,^26^,^27^, are reported in Table 1. Characterization of compounds 1–4 and assay methods can be found in the supporting material.

### Characterization of albumin and liposome nanoparticles

To improve the poor solubility in aqueous solution and the biodistribution of this family of compounds, albumin nanoparticles (AL-1, AL-2, AL-3 and AL-4) and liposomes (LP-1, LP-2, LP-3 and LP-4) were prepared. The albumin-drug nanoparticles, prepared by disulphide-bond induced self-assembly, were analyzed by Dynamic Light Scattering (DLS) and results are reported in Table 2. The mean diameter ranged between 118.8 nm (AL-3) and 165.6 nm (AL-1). This size parameter was associated with high polidispersity indexes (close to 1), which indicated broad size distributions. Indeed, morphological analysis by Field Emission Scanning Electron Microscope (FESEM), confirmed the presence of aggregates (Figure S1). The tendency to form aggregates was also suggested by ζ-potential values belonging to the instability range^44^. The drug loading was around 6% when a very lipophilic compound, namely 1, was encapsulated. However, it rose up to 50% with compounds 2 and 4, both characterized by a better aqueous solubility than compound 1 (Table S1).

Lipid ratio and drug concentration represent two important factors for the encapsulation of drugs in liposomes. Liposomes were prepared by the thin layer evaporation method, using the widely used lipids DPPC:Chol:MPEG-2000-DPPE Na (molar ratio of 20:10:1). The multilamellar vesicles (MLV) were converted by sonication to small unilamellar vesicles (SUV)^45^,^46^ to reduce *in vivo* potential opsonization^47^,^48^. The suspension was filtered through 200 nm filters, to obtain liposomes with a suitable diameter in order to avoid possible occlusion of capillaries *in vivo*^49^. The mean diameter and ζ-potential were measured by DLS (Table 2). The size of nanoparticles ranged between 105 nm and 232 nm (LP-2 and LP-4, respectively), with LP-1 and LP-3 being around 151 nm and 131 nm, respectively. The whole set of liposomes presented good values of ζ-potential (from −27.4 mV to −47.6 mV) and polidispersity index (from 0.2 to 0.4). These data suggested the stability of the final suspension. The concentration of drug (1–4) in each liposomal sample was determined by HPLC-UV-MS analysis, after the disruption of the liposomes. The entrapment efficacy was excellent: above 85% for LP-2, LP-3 and LP-4 and 65% for LP-1. The effects of sonication, temperature and lipid composition were analyzed to maximize the entrapment efficacy and to reduce the variability associated to the solubility of different compounds (Table S2).

### Antiproliferative activity on human cell line SH-SY5Y

The cytotoxicity of nanoparticles (liposomes: LP-1, LP-2, LP-3, LP-4 and albumin: AL-4) was evaluated in SH-SY5Y NB cell line. Taking into account the data regarding albumin nanoparticles previously evaluated, the only sample with satisfactory values of ζ-potential, PDI and EE% values was AL-4. Consequently, it was selected to perform cellular assays in the human NB cell line SH-SY5Y. Pure compounds (DMSO solution), empty liposomes (0.9% NaCl solution, pH 7.4) and albumin (PBS solution, pH 7.4) were included as controls. IC_50_s of these compounds were evaluated at 24, 48 and 72 h, for different concentrations (0.1, 1.0, 10, 50 μM) (Table 3). A greater 24 h-activity was obtained for liposomal samples in comparison with their corresponding drugs. The only exception was pair 3/LP-3 (drug/LP-drug), whose analysis revealed a comparable cytotoxicity. Data collected after 48 h showed a reduced difference in cytotoxicity within each pair. The activity at 72 h was higher for liposomal samples (LP-1 and LP-4) in comparison with the respective free drug (1 and 4). Within the pairs 2/LP-2 and 3/LP-3, a comparable IC_50_ value between each drug and its respective liposomal formulation was found. IC_50_ (72 h) of AL-4 was 12.03 μM, further demonstrating the lower activity of albumin nanoparticles in comparison with the free drug 4 (IC_50_ = 8.63 μM) and the liposome LP-4 (IC_50_ = 1.90 μM).

### Characterization of LP-2

**Figure 2:**
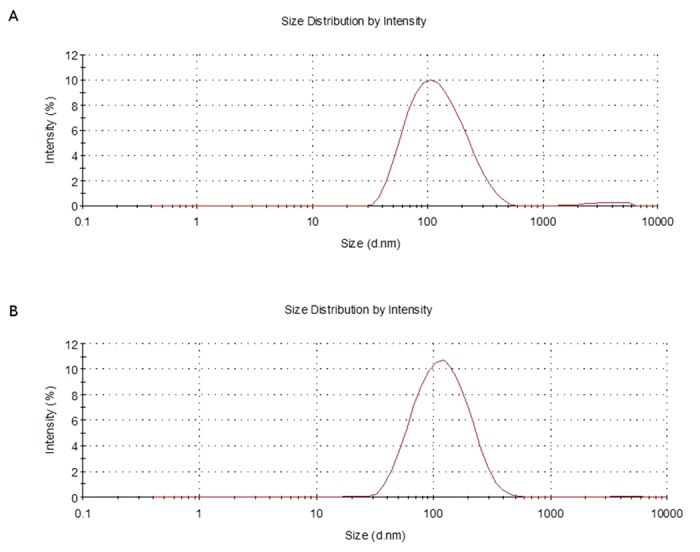
Characterization of size distribution by dynamic light scattering. (**A**) Empty liposomes; (**B**) LP-2.

**Figure 3:**
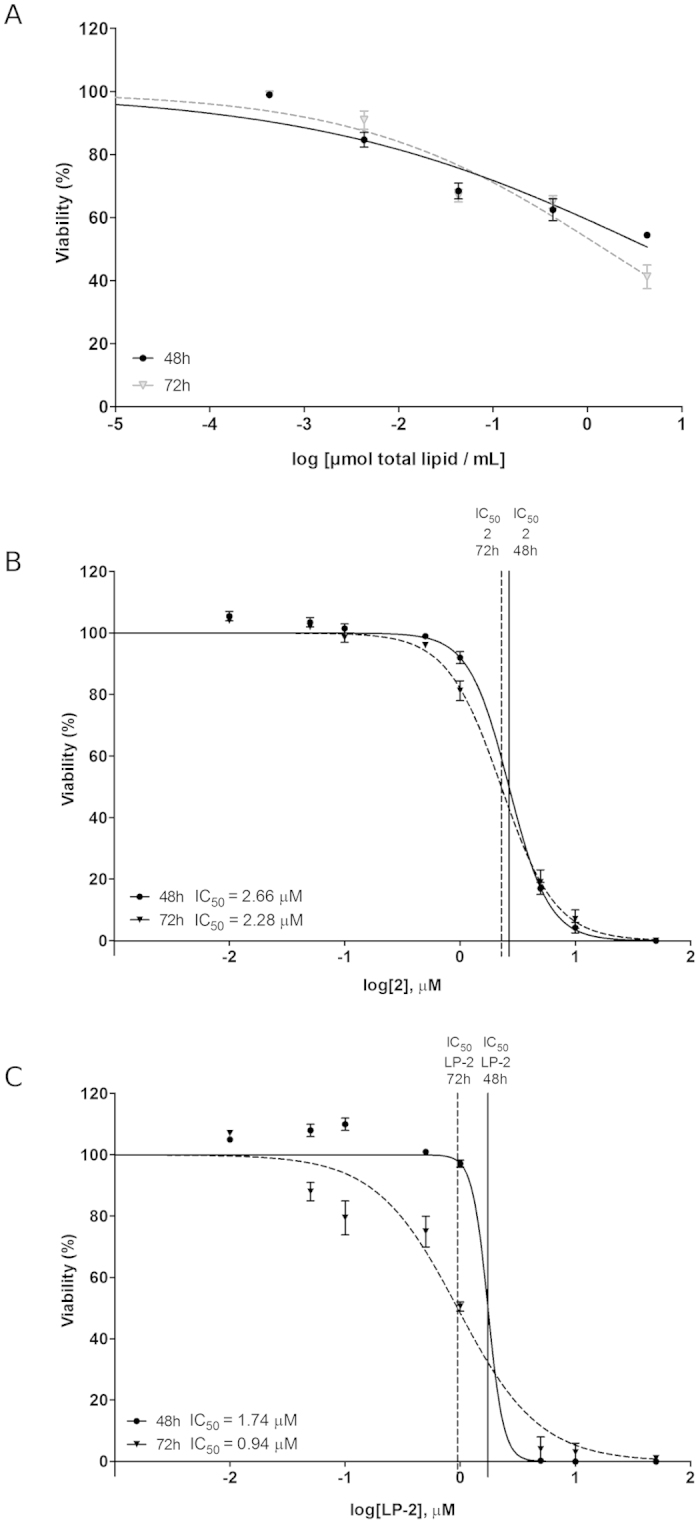
Viability of SH-SY5Y human NB cells evaluated at 48 and 72 h. (**A**) Empty liposomes, (**B**) Free compound 2 and (**C**) LP-2. Compound 2 and LP-2 were tested at the following concentrations: 0.01, 0.05, 0.1, 0.5, 1.0, 10 and 50 μM.

**Figure 4:**
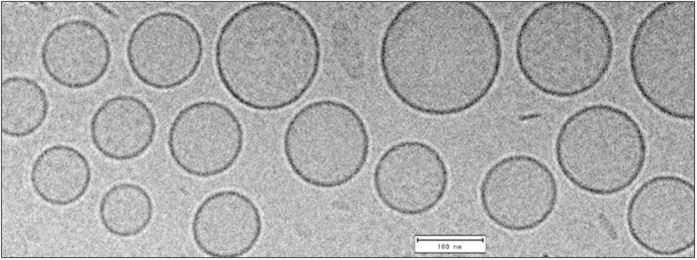
Characterization of LP-2: morphological analysis by Cryo-EM.

As all the other liposomal nanoparticles, LP-2, was characterized by DLS to assess size and ζ-potential and by cellular assays (SH-SY5Y NB) to determine cytotoxicity (Tables 2 and 3). More in detail, Fig. 2 shows the different size distribution by intensity of unloaded liposomes (Fig. 2A) and liposomes loaded with 2 (Fig. 2B). The average size resulted smaller for the sample with encapsulated drug, 105.1 ± 6.39 nm (LP-2) versus 135.2 ± 9.45 nm (Empty LP). Empty liposomes were tested in SH-SY5Y NB cell line in order to exclude any possible cytotoxic activity (Fig. 3A). This was particularly important because, although DPPC and DPPE are widely used to produce liposomes, recent studies^50^ demonstrated that saturated fatty acids might have a toxic effect in NB cells. In our assays, however, the obtained curve confirmed that liposomes do not influence significantly cell viability, indicating that the amount of saturated fatty acids derived from the liposomal preparation is not enough to determine a cytotoxic effect. LP-2 demonstrated an activity comparable with the one of compound 2 at 48 h (Fig. 3B). Additionally, the liposomal formulation at 72 h showed an enhanced cytotoxic effect (Fig. 3C). Subsequently, liposomal preparation LP-2 was morphologically analyzed by cryo-Electron Microscopy (cryo-EM) (Fig. 4). The result confirmed the presence of a homogeneous population of unilamellar nanoparticles with size around 100 nm. Liposomes were round, smooth and free from drug crystals; while bilamellar, multilamellar or giant-liposomes were not observed in the image from cryo-EM. The average thickness of the phospholipidic bilayer corresponded to 5.77 ± 1.05 nm (12 measurements performed by ImageJ Software, 1.46r).

### Delivery of liposomes in neuroblastoma cells

**Figure 5:**
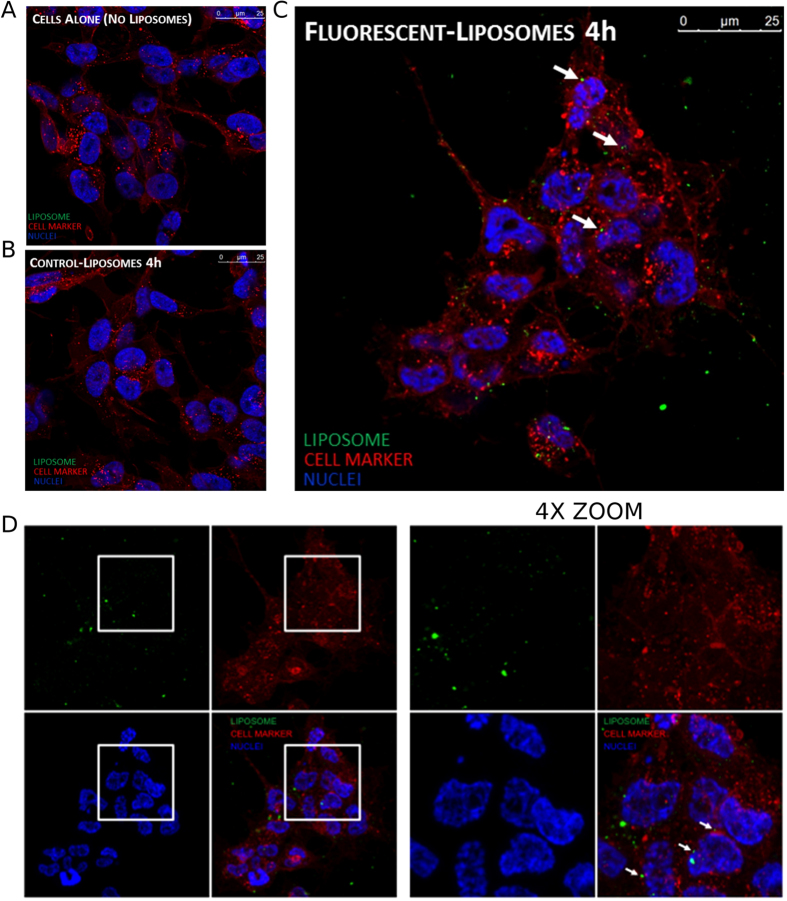
Confocal microscopy experiments. Neuroblastoma cells (SH-SY5Y) were seeded on glass coverslip and then incubated for 4 h with: (**A**) control, (**B**) normal liposomes, (**C**) fluorescent liposomes. Liposomes are visualized in green, cellular membranes in red and nuclei in blue. (**D**) Z-stack projection, on the right 4X zoom of the highlighted regions of the left panel.

Neuroblastoma cells were incubated with normal and fluorescent liposomes to evaluate their possible uptake. Fluorescent liposomes were prepared incorporating a fluorescent phospholipid (NBD-DOPE) in the lipid mixture. After 4 h of incubation, fluorescent liposomes were internalized into neuroblastoma cells (Fig. 5A,B). Z-stack image confirmed the localization of NBD-labelled liposome inside SH-SY5Y cells (Figure S2).

### *In vitro* release

**Figure 6:**
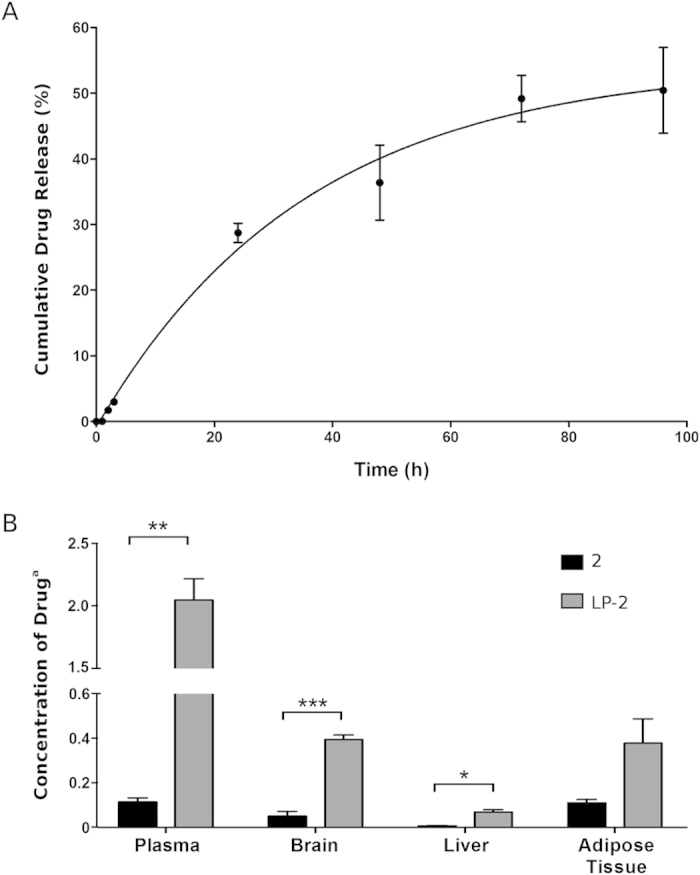
*In vitro* release and biodistribution at 24 h. (**A**) Release of compound 2 from liposomal system LP-2 in physiological conditions, with 50 mg/mL of BSA, at 37 °C. (**B**) Concentration of compound 2 determined in plasma, brain, liver and adipose tissue, after the administration of the free drug 2 (black) and the liposomal formulation LP-2 (grey). ^a^The concentration is expressed as μg/mL for plasma and as μg/g for brain, liver and adipose tissue.

To determine the stability in physiological settings and to confirm the release of the drug 2 from its liposomal formulation LP-2, the release kinetics of 2 was analysed *in vitro* by measuring the concentration of drug released from liposomes into a physiological medium (BSA 50 mg/mL) at 37 °C (Fig. 6A). The cumulative percentage of drug release was determined over a 96 h-period. The results demonstrated the stability of the sample in physiological conditions at 37 °C. In fact, the percentage of 2 released from LP-2 resulted below 28% after 24 h and 49% over a 72 h-period. The final percentage of drug released was of 50.5%. In addition, the rate of drug release was evaluated during the 24 h (0.96 μg/h).

### Biodistribution at 24 h

Biodistribution of LP-2 and free drug 2 were evaluated in male Sprague-Dawley rats. The concentration of compound 2 was determined after 24 h in the following tissues: plasma, brain, liver and adipose tissue (Fig. 6B). The concentration of the active compound was one order of magnitude higher in the plasma of rats treated with LP-2, validating the use of liposomes to enhance the plasma-exposure of a drug. In fact, the concentration of the free compound 2 was 0.11 μg/mL and 2.05 μg/mL in the groups treated with 2 and LP-2 respectively. The concentration of compound recovered in the brain was 0.05 μg/g (group treated with 2) versus 0.39 μg/g (group treated with LP-2). Again, the increase of quantity of compound 2 indicated the improved biodistribution of 2 when liposomes are used as drug delivery systems.

## Discussion

With the aim determining if the use of albumin nanoparticles and liposomes could represent a possible strategy to improve pharmacokinetic properties of our compounds, four pyrazolo[3,4-*d*]pyrimidines (1–4), selected from our library of compounds, were encapsulate in these nanosystems. Pyrazolo[3,4-*d*]pyrimidines have demonstrated a high anticancer activity associated to the inhibition of tyrosine kinases c-Src and Bcr-Abl and we have recently demonstrated that pyrazolo[3,4-*d*]pyrimidines were able to reduce tumor growth in NB xenograft mouse model^25^. Thus, we have decided to focus on NB for this study. Compounds 1–4 were selected on the basis of previously reported data regarding activity and *in vitro* ADME properties^25^,^26^,^27^. Nanoparticles were characterized by DLS regarding their size, polydispersity index and ζ-potential. Particle size has a significant impact on the circulation time^51^. Furthermore, the dimensions of the smallest capillaries need to be taken into account to avoid a possible obstruction. Particle size also affects cellular uptake, influencing phagocytosis and endocytosis. In general, the larger is the nanoparticle, the faster is the clearance by the MPS. Optimal size to facilitate extravasation is about 150 nm or less, i.e. Doxil^©^ has size between 80–100 nm and Myocet^©^ is around 150 nm. In this context, this study demonstrated that our liposomes are suitable drug-delivery systems with a diameter that ranges from 105 nm to 232 nm. Another important feature for nanoparticle dispersion stability is the ζ-potential that indicates the degree of electrostatic repulsion between particles. In detail, nanoparticles with ζ-potential values greater than + 25 mV or less than −25 mV typically have high degrees of stability^44^. Showing a ζ-potential value between −28.65 mV and −48.00 mV, our liposome systems were confirmed to be stable.

On the other side, albumin systems were characterized by ζ-potentials and PDIs into the range of instability (values around −10 mV and close to 1, respectively). These data suggested that these nanoparticles might form aggregates (hypothesis confirmed by DLS analysis and FESEM study). However, we decided to proceed with the evaluation of the cytotoxicity of the most promising albumin sample, namely AL-4.

Liposomes demonstrated greater 24 h-activity when compared with the appropriate free drug (comparable IC_50_s of LP-3 and 3, represented the only exception). This difference in cytotoxicity might be related to different internalization processes. In fact, while the free drug can enter into cells by passive membrane transport, liposomes need to be taken up by different processes (i.e. endocytosis, fusion). At 48 h and 72 h, pairs LP-2/2 and LP-3/3 showed similar IC_50_s. However, LP-1 and LP-4 demonstrated higher cytotoxicity than the respective free drug both, in 48 h and 72 h measurements. Cellular assays further demonstrated the unfavourable properties of albumin nanoparticles, as the IC_50_ value was higher than the ones obtained by testing free drug 4 and liposomes LP-4. Given their poor properties (PDI, ζ-potential, EE% and IC_50_) albumin nanoparticles were not further characterized.

LP-2 resulted our most promising liposomal candidate because of the following: (I) IC_50_ value of 0.97 μM against NB cells, (II) 99% of entrapment efficiency, (III) ζ-potential value of −39.9 ± 0.55 mV and (IV) size of 105.1 ± 6.39 nm. Additionally, 2 was the compound previously tested in NB xenograft mouse model^25^. Thus, LP-2 was further characterized by cryo-EM, confirming the data obtained by DLS analysis. The sample was indeed characterized by a homogenous population of unilamellar liposomes with size around 100 nm. Furthermore, a confocal microscopy-based study was performed to elucidate whether the drug is released from liposomes outside the cells or inside the cytoplasm, after an internalization of the whole liposomal system. The analysis showed the presence of fluorescent liposomes into the cytoplasm of NB cells, suggesting a delivery of the free drug after cellular uptake of liposomes.

The release of compound 2 from the liposomal system LP-2 was evaluated with an *in vitro* assay that allowed the quantification of compound 2 likely to be released from liposomes into physiological conditions. The resulting monophasic release curve was a typical feature of unilamellar liposomes, accordingly with DLS and cryo-EM results. A proof of concept *in vivo* experiment was then performed to evaluate and compare the biodistribution of free compound 2 and liposomes LP-2 in several tissues, at 24 h. Additionally, this study was important to determine the *in vivo* stability of our liposomal systems. Data showed a generally improved biodistribution - higher concentration - of the drug 2 when liposomes were used as drug-delivery system. Considerably, when LP-2 was administered a 20-fold increase in plasma-concentration of free compound 2 was observed. In addition, the quantity of compound 2 recovered from the brain tissue after injection of LP-2 was significantly higher than the concentration obtained after administration of compound 2.

In conclusion, this study validated the use of stealth liposomes to improve the biodistribution of pyrazolo[3,4-*d*]pyrimidines. Herein we demonstrate that liposomes retain the efficacy of our encapsulated drug against SH-SY5Y NB cells and that this cytotoxic activity is likely to be exerted by the release of the active compound in the cytoplasm after uptake of the nanoparticles. Importantly, the liposomal system resulted stable and able to release the encapsulated drug in physiological conditions and the biodistribution assay finally proved the beneficial properties of LP-2. Further preclinical *in vivo* studies will allow the determination of the full pharmacokinetic profile and the therapeutic efficacy of this novel formulation.

## Methods

### Materials

Pyrazolo[3,4-*d*]pyrimidine compounds (1–4), were previously synthesized and characterized by our research group^25^,^26^,^27^. 1,2-Dipalmitoyl-*sn*-glycero-3-phosphocholine semisynthetic, ≥99% (DPPC), *N*-(carbonyl methoxypolyethylenglycol 2000)-1,2-dipalmitoyl-*sn*-glycero-3-phosphoethanolamine sodium salt (MPEG-2000-DPPE Na), 1,2-dioleoyl-sn-glycero-3-phosphoethanolamine,7-nitrobenzofurazan-labeled (NBD-DOPE), 4′,6-diamidino-2-phenylindole (DAPI), cholesterol (Chol), human serum albumin (HSA) (lyophilized, 97%), dialysis tubing cellulose membrane (cut-off 14 kDa), L-cysteine and and D,L-glyceraldehyde and all the solvents were purchased from Sigma Aldrich. Nylon syringe filters with pores of 0.2 μm (Acrodisc 13 mm Syringe Filter) and PTFE syringe filters with pores of 0.2 μm (Acrodisc 13 mm Syringe Filter) were from purchased VWR. High purity deionised water was obtained from milli-Q (Millipore, Milford, MA, USA). Wheat germ agglutinin, Alexa Fluor 647 Conjugate (WGA) was purchased from Life Technologies. Fetal Bovine Serum (FBS), Dulbecco’s Modified Eagle Medium: Nutrient Mixture F-12 (DMEM/F12), L-glutamine (L-Glu) and Penicillin-Streptomycin (Pen/Strep) were purchased from Euroclone.

### Preparation of albumin-drug nanoparticles

The albumin nanoparticles were prepared by mixing 100 μL of compound solution in EtOH (1 mg/mL) with 900 μL of HSA solution in PBS (pH 7.4, 25 mM) (5 mg/mL)^52^. Cysteine was added up to a final concentration of 5 mg/mL, and the resulting solution was kept at 37 °C, for 45 min. The excess of cysteine was eliminated by dialysis using a cellulose membrane (capacity 60 mL/ft, cut-off 14 kDa, diameter 16 mm, width 25mm). D,L-glyceraldehyde was added to this solution, to a final concentration of 5 μM^53^. The sample was then filtered through a nylon syringe filter with pores of 0.2 μm (Acrodisc 25 mm Syringe Filter) to obtain a sterile solution of the drug-albumin nanoparticles. The concentration of the compound in the final sample was determined by HPLC-UV-MS (HPLC-UV-MS method in the supporting material), after denaturation of the protein by acetonitrile and centrifugation (4500 rpm, 15 min, 4 °C).

### Preparation of Liposome-drug nanoparticles

The method developed by A.D. Bangham, namely thin layer evaporation, was used for the preparation of LP-1, LP-2, LP-3 and LP-4. Lipids DPPC, Chol and MPEG-2000-DPPE Na (molar ratio 20:10:1) were dissolved in a mixture of CHCl_3_:MeOH = 3:1 (3 mL, round-bottomed flask). A 1 mM solution of each compound (1, 2, 3, 4) in CHCl_3_:MeOH = 3:1 was prepared. An aliquot was transferred in the lipid solution to obtain 500 μM as final concentration of compound. The solvent was removed under reduced pressure to obtain a thin layer of lipids (kept under high vacuum overnight). The thin layer was hydrated with a 0.9% NaCl solution and the mixture was stirred for 1 hour at a temperature higher than the Tm of the lipids. The suspension was mixed (Vortex, 3 min) and SUV liposomes were formed by sonication for 20 min at room temperature. The solution was filtered with PTFE syringe filters with pores of 0.2 μm (Acrodisc 13 mm Syringe Filter), to eliminate liposomes with diameter over 200 nm and potentially precipitated drugs. The drug concentration was determined after disruption of liposomes by treatment with ethanol at 40 °C (1:1 = v/v). The compound was extracted and quantified by HPLC-UV-MS (HPLC-UV-MS method in the supporting material).

### Measurements of size and ζ-potential

Size distribution and ζ-potential were determined by Dynamic Light Scattering (DLS) (Zeta Sizer Nano ZS90, Malvern Instruments Ltd, Malvern, UK). Measurements were performed directly after rehydration of the samples with 1 mM NaCl aqueous solution. Samples stability was evaluated by DLS after one week.

### Antiproliferative activity on human cell line SH-SY5Y

*In vitro* experiments were carried out using the human NB cell line SH-SY5Y. Cells were purchased from American Type Culture Collection (ATCC, Manassas, VA, USA) and were cultured in DMEM/F12 1:1 medium supplemented with 10% FBS, 2 mM L-glutamine and 10000 units/mL Penicillin/Streptomycin at 37 °C in 5% CO_2_ atmosphere. In order to determine the antiproliferative effect of drugs (1–4) and nanosystems, SH-SY5Y cells were seeded at 10^5^ cells/well density and treated with albumin (PBS solution, pH 7.4) or empty liposomes (0.9% NaCl solution, pH 7.4), compounds encapsulated with albumin (PBS solution, pH 7.4) or liposomes LP-1, LP-3 and LP-4 (0.9% NaCl solution, pH 7.4) and free compounds 1, 3 and 4 (DMSO solution) at increasing concentrations (0.1 μM, 1.0 μM, 10 μM and 50 μM). IC_50_ determination for LP-2 and compound 2 was performed testing the following concentrations as well: 0.01 μM, 0.05 μM, 0.5 μM and 5 μM. The cultures were maintained at 37 °C in 5% v/v CO_2_ for 24, 48 and 72 h. Cell number and viability were evaluated using the Bürker chamber, after treatment with Trypan Blue. IC_50_ was calculated by GraphPad Prism 6.0 software using the best fitting sigmoid curve.

### Morphology study

Morphology was observed by Cryo Electron Microscopy. 3 μL of sample were applied on Quantifoil^®^ holey carbon grids (copper Multi A, Quantifoil^®^ Micro Tools GmbH, Jena, Germany). Excess fluid was blotted from the grid for 2 sec with Whatman filter paper and then plunge frozen in liquid ethane, using a plunge freezer, to achieve sample vitrification. Frozen samples were stored in liquid nitrogen until EM imaging. Vitrified samples were imaged using a CM200 FEG transmission EM (FEI, Eindhoven, the Netherlands) operated at 200 keV and equipped with a F224HD 2048 × 2048 CCD camera (TVIPS Gauting, Germany). EM images were acquired at 27,500× magnification (pixel size 0,602 nm) at −12, −18 μm defocus.

### Confocal microscopy studies on the cellular uptake

For confocal microscopy experiments, fluorescent liposomes, carrying NBD-DOPE in the lipid bilayer, were used. DPPC, Chol and MPEG-2000-DPPE were dissolved at a molar ratio of 20:10:1, respectively, CHCl_3_:MeOH = 3:1 v/v. Then, NBD-DOPE was added to the lipid mixture to a final molar ratio of 2% of total lipids. The lipid mixture was dried and placed in a vacuum desiccator for at least 24 h to ensure complete solvent removal. Finally, lipid film was rehydrated with NaCl 0.9% solution to obtain a NBD-DOPE final concentration of 440 μM. SH-SY5Y cells were maintained in DMEM/F12 1:1 medium supplemented with 10% FBS, 2 mM L-glutamine and 10000 units/mL Penicillin/Streptomycin at 37 °C in 5% CO_2_ atmosphere. SH-SY5Y cells were plated on glass coverslip at a density of 20000 cells per well in 24-well plates. After 24 h, cells were incubated with liposome formulation, and then fixed in 4% paraformaldehyde in PBS for 20 min. Nuclei and cellular membranes were stained respectively with a 6 μM solution of DAPI and 2 μg/mL WGA Alexa Fluor 647 labelled for 10 min. Coverslips were mounted in fluorescence mounting medium (Dako, S3023). Samples were visualized on a TSC SP5 confocal microscope (Leica, 5100000750) installed on an inverted LEICA DMI 6000CS (10741320) microscope and equipped with an oil immersion Plan Apo 63 × 1.4 NA objective. Images were acquired using the LAS AF acquisition software (Leica, 10210). NBD fluorescence was acquired in 500–560 nm range and excited with 476 nm argon laser, DAPI was acquired in 420–480 nm range and excited with 405 nm UV laser and WGA was acquired in 650–730 nm range and excited with 633 nm laser.

### *In vitro* release

Rates of drug release were studied by dialyzing the nanosystem LP-2 (3.5 mL, 0.4 mM) against PBS (20 mL, pH 7.4, 10 mM) with 50 mg/mL of BSA (physiological plasma concentration). The entire system was stirred at 37 °C and samples (1 mL, from the PBS-BSA) were collected at different time points (0, 1, 2, 3, 24, 48, 72 and 96 h) (1 mL of PBS-BSA was added each time to maintain sink condition). Each sample was treated with 1 mL of ACN and centrifuged at 4000 rpm for 20 min. Then, the supernatant was recovered, concentrated under reduced pressure and analyzed to determine the concentration of compound 2 by HPLC-UV-MS (HPLC-UV-MS method in the supporting material).

### *In vivo* plasmatic distribution

Male Sprague Dawley rats (Charles River, Milan, Italy) were maintained according to ethical EEC regulations for animal research. The animal protocols used were reviewed and approved by the Animal Care and Ethics Committee of the University of Siena, Italy. The animals were anesthetized (*i.p.* xylazine hydrochloride, 10 mg/kg, Xilor, Bayer AG, and ketamine hydrochloride 35 mg/kg, Ketavet, Gellini) and treated by *i.v.* injection through the tail vein. The experiment was performed in triplicate. The two groups received intravenously LP-2 (500 μL, PBS, pH 7.4) or 2 (50 μL in DMSO), corresponding to a bolus of 10 mg/Kg. After 24 h, animals were sacrificed and blood and tissues were treated for the quantitative analysis. The blood, previously heparinized, was centrifuged at 4000 rpm for 20 min to separate the plasma fraction and then, 500 μL were collected in a test tube. Tissues were homogenized using a glass/glass Potter-Elvehjem homogenizer. For each sample 1 mL of ACN (in the presence of the internal standard 1-(2-chloro-2-(4-chlorophenyl)ethyl)-*N*-(3-fluorobenzyl)-1*H*-pyrazolo[3,4-*d*]pyrimidin-4-amine, 10 μM) was added to denature proteins and to extract 2. Samples were centrifuged at 4000 rpm for 20 min; the supernatant was recovered, evaporated and analyzed by HPLC-UV-MS (HPLC-UV-MS method in the supporting material).

## Additional Information

**How to cite this article**: Vignaroli, G. *et al.* Improvement of pyrazolo[3,4-*d*]pyrimidines pharmacokinetic properties: nanosystem approaches for drug delivery. *Sci. Rep.* **6**, 21509; doi: 10.1038/srep21509 (2016).

## Supplementary Material

Supplementary Information

## Figures and Tables

**Table 1:** Compounds 1–4: Activity against Src and ADME properties^a^.

Cpd	Aqueous Solubility (μg/mL)	LogP^b^	Src (K_*i*_ μM)	PAMPA (10^−6^ cm^2^/sec)	MR^c^ (%)	Metabolic Stability^d^ (%)
1	0,21	6,555	3.7	3.98	46.8	95.8
2	3,71	5,813	0.13	5.27	46.1	96.0
3	0,22	5,678	0.4	5.51	48.9	97.9
4	0,90	5,992	0.01	4.53	49.6	93.5

^a^All the data were previously reported, see supporting material for experimental details^25^,^26^,^27^.

^b^Calculated by Qikprop.

^c^Membrane Retention (MR) expressed as percentage of compound unable to reach the acceptor compartment.

^d^Expressed as percentage of unmodified drug.

**Table 2:** Properties of liposomes and albumin nanoparticles.

Formulation	Size^a^ (nm)	Polydispersity index^a^ (PDI)	Entrapment Efficiency^b^ (%)	ζ-potential^a^ (mV)
Liposomes	135.2 ± 9.45	0.23 ± 0.01	–	−27.4 ± 1.79
LP-1	151.3 ± 2.06	0.40 ± 0.04	65.0 ± 6.36	−41.9 ± 5.75
LP-2	105.1 ± 6.39	0.21 ± 0.01	99.1 ± 0.71	−39.9 ± 0.55
LP-3	131.3 ± 5.14	0.21 ± 0.03	85.2 ± 9.84	−28.8 ± 0.15
LP-4	232.4 ± 7.35	0.13 ± 0.01	96.3 ± 2.83	−47.6 ± 0.38
Albumin	9.30 ± 0.15	0.26 ± 0.01	–	−10.3 ± 1.41
AL-1	165.6 ± 6.11	0.97 ± 0.01	6.7 ± 0.91	−15.2 ± 1.17
AL-2	125.3 ± 3.76	0.68 ± 0.01	51.0 ± 2.88	−4.47 ± 1.93
AL-3	118.8 ± 3.00	0.58 ± 0.06	24.8 ± 1.23	−13.4 ± 2.90
AL-4	135.4 ± 15.9	0.55 ± 0.01	52.3 ± 1.82	−14.9 ± 1.46

^a^Determined by DLS (Nano-Zeta Sizer, Malvern Instruments Ltd, Malvern, UK).

^b^For experimental details, see supporting material.

**Table 3:** Cytotoxicity in SH-SY5Y NB cells.

Cpd/Formulation	IC_50_ 24 h (μM)	IC_50_ 48 h (μM)	IC_50_ 72 h (μM)
1	21.84 ± 1.70	16.5 ± 1.67	13.54 ± 2.00
LP-1	8.03 ± 0.59	7.14 ± 1.90	6.35 ± 1.44
2	12.6 ± 1.6	2.66 ± 0.015	2.28 ± 0.29
LP-2	6.80 ± 0.98	1.74 ± 0.31	0.94 ± 0.68
3	3.04 ± 0.09	2.65 ± 0.08	1.54 ± 1.07
LP-3	3.50 ± 0.17	1.52 ± 0.94	1.54 ± 0.79
4	13.64 ± 0.94	10.06 ± 0.50	8.63 ± 0.75
LP-4	3.87 ± 0.51	1.91 ± 1.46	1.90 ± 0.46
AL-4	20.04 ± 1.04	12.02 ± 0.97	12.03 ± 0.30

SH-SY5Y cells were seeded at 10^5^ cells/well density. The cultures were maintained at 37 °C in 5% v/v CO_2_ for 24, 48 and 72 h. IC_50_s were evaluated by Trypan blue assay and calculated by GraphPad Prism 6.0 software using the best fitting sigmoid curve.
