# Supplementary material for: Improvement of pyrazolo[3,4-d]pyrimidines pharmacokinetic properties: nanosystem approaches for drug delivery
Source: Sci Rep. 2016 Feb 22;6:21509. doi: 10.1038/srep21509 (PMC4761914; doi:10.1038/srep21509)
Supplement: Supplementary Information [file srep21509-s1.pdf]

Improvement of pyrazolo[3,4-*d*]pyrimidines pharmacokinetic properties: nanosystem approaches for drug delivery

Giulia Vignaroli[1,2,*], Pierpaolo Calandro[1,*], Claudio Zamperini[1,2], Federica Coniglio[1,2], Giulia Iovenitti[1,2], Matteo Tavanti[1], David Colecchia[3], Elena Dreassi[1], Massimo Valoti[4], Silvia Schenone[5], Mario Chiariello[3], Maurizio Botta[1,2,6].

[1] Dipartimento Biotecnologie, Chimica e Farmacia, Università degli Studi di Siena, via Aldo Moro 2, 53100, Siena (IT)
[2] Lead Discovery Siena S.r.l., via Vittorio Alfieri 31, 53019, Castelnuovo Berardenga, Siena (IT)
[3] Consiglio Nazionale delle Ricerche, Istituto di Fisiologia Clinica and Istituto Toscano Tumori, Core Research Laboratory, Via Fiorentina 1, 53100, Siena.
[4] Dipartimento di Scienze della Vita, Università degli Studi di Siena, via Aldo Moro 2, 53100, Siena (IT)
[5] Dipartimento di Farmacia, Università di Genova, Viale Benedetto VX 3, 16132, Genova (IT)
[6] Biotechnology College of Science and Technology, Temple University, Biolife Science Building, Suite 333, 1900 N 12th Street, Philadelphia, Pennsylvania 19122.

*These authors contributed equally to this work.

Correspondence and requests for materials should be addressed to M.B. (email: *botta@unisi.it*)

Supporting Material

1) *Pyrazolo[3,4-d]pyrimidine compounds (**1- 4**) characterization* **3**

2) *HPLC-UV-MS method* **3**

3) *ADME assays…………………………………………………………………………………………………* **4**

4) *FESEM analysis of albumin nanoparticles…………………………………………………….* **6**

5) *Albumin-drug nanoparticles preparation……………………………………………………* **6**

6) *Entrapment Efficacy (EE%)……………………………………………………………………….* **7**

7) *Liposome-drug nanoparticles preparations…………………………………………………* **7**

8) *Z-stack projection of SH-SY5Y cells incubated with fluorescent liposomes………* **8**

1) *Pyrazolo[3,4-d]pyrimidine compounds (1- 4) characterization*

***N*-benzyl-1-(2-chloro-2-phenylethyl)-6-(methylthio)-1*H*-pyrazolo[3,4-*d*]pyrimidin-4-amine (1).** [Navarra, M. *et al. BMC Cancer* **2010**, *10*, 602-614.]

White solid, yield 81%. ^{1}H NMR ($CDCl_3$): δ 2.58 (s, 3H, CH_3), 4.70-4.94 (m, 4H, CH_2N + CH_2NH), 5.50-5.60 (m, 1H, CHCl), 7.24-7.96 (m, 10H Ar), 7.72 (s, 1H, H-3). MS (*m/z):* [M+1]$^+$ 410. Anal. ($C_{21}H_{20}N_5SCl$) C, H, N, S.

***N*-(3-Bromophenyl)-1-(2-chloro-2-phenylethyl)-6-[(2-morpholin-4-ylethyl)thio]-1*H*-pyrazolo[3,4-*d*]pyrimidin-4-amine (2).** [Tintori, C. *et al. J. Med. Chem.* **2015**, *58*, 347-361.]

White solid, yield 61%. ^{1}H NMR ($CDCl_3$): δ2.90-3.99 (m, 12H, 4CH_2 morph. + CH_2N+ CH_2S), 4.63-4.85 and 5.04-5.21 (2m, 2H, CH_2N pyraz), 5.55-5.70 (m, 1H, CHCl), 7.03-8.52 (m, 10H, 9 Ar + H-3), 11.33 (br s, 1H, NH disappears with D_2O). MS (*m/z):* [M+1]$^+$ 575. Anal. ($C_{25}H_{26}N_6OBrClS$) C, H, N, S.

***N*-(3-Chlorophenyl)-6-[(2-morpholin-4-ylethyl)thio]-1-(2-phenylpropyl)-1*H*-pyrazolo[3,4-*d*]pyrimidin-4-amine (3).** [Tintori, C. *et al. J. Med. Chem.* **2015**, *58*, 347-361.]

Pale yellow solid, yield 52%.^{1}H NMR ($(CD_3)_2SO$): δ 1.23 (d, *J* = 7.0 Hz, 3H, CH_3), 2.52-2.67, 2.74-2.81, 3.24-3.40 and 3.43-3.59 (4m, 8H, 2CH_2N morph. + SCH_2CH_2), 3.65-3.80 (m, 4H, 2CH_2O morph.), 3.85-3.90 (m, 1H, $\underline{CH}CH_3$), 4.40-4.50 (m, 2H, CH_2N pyraz.), 7.20-7.40 (m, 9H Ar), 7.97 (s, 1H, H-3), 10.40 (br s, 1H, NH disappears with D_2O). MS (*m/z):* [M+1]$^+$ 510. Anal. ($C_{26}H_{29}N_6OClS$) C, H, N, S.

3-{[1-(2-Chloro-2-phenylethyl)-6-(isopropylthio)-1*H*-pyrazolo[3,4-*d*]pyrimidin-4-yl]amino}phenol (4). [Tintori, C. *et al. J. Med. Chem.* **2015**, *58*, 347-361.]

Light brown solid, yield 77%.^{1}H NMR ($CDCl_3$): δ 1.46 (d, *J* = 6.0 Hz, 3H, CH_3), 1.49 (d, *J* = 6.0 Hz, 3H, CH_3), 3.97-4.07 (m, 1H, SCH), 4.70-4.75 and 4.83-4.88 (2m, 2H, CH_2N) 5.48-5.51 (m, 1H, CHCl), 6.78-6.80, 6.94-7.02 and 7.21-7.41 (3m, 10H, 9 Ar + H-3). MS (*m/z):* [M+1]$^+$ 441. Anal. ($C_{22}H_{22}N_5ClOS$) C, H, N, S.

2) *HPLC-UV-MS method*

A HPLC-UV-MS system was used for quantitative analysis. LC analysis were performed by Agilent 1100 LC/MSD VL system (G1946C) (Agilent Technologies, Palo Alto, CA) constituted by a vacuum solvent degassing unit, a binary high-pressure gradient pump, a 1100 series UV detector and a 1100 MSD model VL benchtop mass spectrometer. The Agilent 1100 series mass spectra detection (MSD) single-quadrupole instrument was equipped with the orthogonal spray API-ES (Agilent Technologies, Palo Alto, CA). Nitrogen was used as nebulizing and drying gas. The pressure of the

nebulizing gas, the flow of the drying gas, the capillary voltage, the fragmentor voltage and the vaporization temperature were set at 40 psi, 9 L/min, 3000 V, 70 V and 350 °C, respectively. UV detection was monitored at 280 nm. The HPLC-ESI-MS determination was performed by operating the MSD in the positive ion mode. Spectra were acquired over the scan range m/z 50-1500 using a step size of 0.1 u. Chromatographic analysis were performed using a Phenomenex Kinetex C18-100A column (150 x 4.6 mm, 5 µm particle size) at room temperature. Analysis were carried out using gradient elution of a binary solution; (eluent A: ACN, eluent B: Water). The analysis started with 0% of A (from t = 0 to t = 3 min), then A was increased to 98% (from t = 3 to t = 12 min), then kept at 98% (from t = 12 to t = 18 min). The analysis were performed at a flow rate of 0.8 mL/min with 20 µL as injection volume. The quantification of all compounds was calculated by referring to the appropriate calibration curves in methanol.

3) ADME assays

Aqueous Solubility

Each solid compound (1 mg) was added to 1 mL of H_2O. The samples were shaken in a shaker bath at 20 °C for 24 hrs. The suspensions were filtered through a 0.45-µm nylon filter (Acrodisc), and the concentration of solubilized compounds were determined by UV/LC-MS (the determination of each compound was performed in triplicate).

Quantification of the single compound was made by comparison with apposite calibration curves realized with standard solutions in methanol.

Parallel Artificial Membrane Permeability Assay (PAMPA)

Donor solution (0.5 mM) were prepared by diluting 1 mM DMSO compound stock solution using phosphate buffer (pH 7.4, 0.025 M). Filters were coated with 5 µL of a 1% (w/v) dodecane solution of L-α-phosphatidylcholine. Donor solution (150 µL) were added to each well of the filter plate. To each well of the acceptor plate were added 300 µL of solution (50% DMSO in phosphate buffer). All compounds were tested in three different plates in different days. The sandwich was incubated for 5 hours at room temperature under gentle shaking. After the incubation time, the sandwich plates were separated and samples taken from both receiver and donor sides were analysed using LC-UV-MS method. Permeability (P_{app}) for PAMPA, was calculated according to the following equation,

obtained from Wohnsland and Faller and Sugano *et al.* equation with some modification in order to obtain permeability values in cm s^{-1}:

$$P_{app} = \frac{V_D V_A}{(V_D + V_A)At} - \ln(1 - r)$$

where V_A is the volume in the acceptor well (cm^3), V_D is the volume in the donor well (cm^3), A is the "effective area" of the membrane (cm^2), t is the incubation time (s) and r the ratio between drug concentration in the acceptor and equilibrium concentration of the drug in the total volume (V_D+V_A). Drug concentration is estimated by using the peak area integration.
Membrane Retention (MR) was calculated according to the following equation:

$$\%MR = \frac{r - (D + A)}{C} 100$$

Where r is the ratio between drug concentration in the acceptor and equilibrium concentration of the drug in the total volume (V_D+V_A), D, A and C represent the drug concentration in donor and, acceptor compartment respectively, C is the equilibrium concentration.

Metabolic Stability in HLM (Human Liver Microsomes)
Each compounds in MeOH solution was incubated at 37 °C for 60 min in 0,025 M phosphate buffer (pH 7.4), 5µL of human liver microsomal protein (0.2 mg mL^{-1}), in the presence of a NADPH-generating system at final volume of 0.5 mL (compounds' final concentration 50 µM). The reaction was stopped by cooling in ice and adding 1.0 mL of acetonitrile. The reaction mixtures were then centrifuged and the parent drug and metabolites were subsequently determined by UV/LC-MS. Chromatographic analysis were performed with the UV/LC-MS method above describe.
The percentage of not metabolized compound was calculated by comparison with reference solutions.

4) *FESEM analysis of albumin nanoparticles*

Albumin nanoparticles were analyzed by field emission scanning electron microscope (FESEM SIGMA VP Zeiss, Germany) equipped by a energy dispersive X-ray spectroscopy system with an In-Lens detector. Samples were diluted in water and a few drops were placed on carbon-filmed grids and introduced in the sample holder stub. Samples were dried and analyzed using an accelerating voltage between 10 and 20 kV.

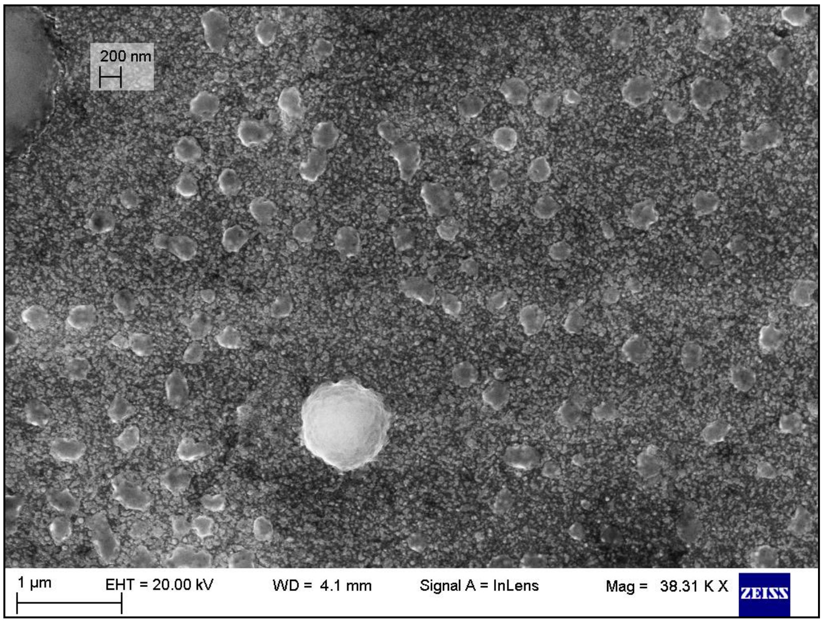

Figura S1: Albumin nanoparticles morphology study by FESEM.

5) *Albumin-drug nanoparticles preparation*

In order to define the best experimental conditions to obtain albumin nanoparticles, different experiments were performed, varying several parameters (concentration of HSA, drug concentration, ratio HSA/Cys). Table S1 shows some representative experiments regarding our best albumin candidate **AL-4**. The hydration volume was kept fixed at a value of 5 mL. To the optimized preparation column (2), D,L-glyceraldehyde was added as cross-linking agent.

Table S1.											
**Batch. No.**	**(1)**	**(2)**	**(3)**	**(4)**	**(5)**	**(6)**	**(7)**	**(8)**	**(9)**	**(10)**	**(11)**
**[HSA] mg/mL**	5	5	5	5	5	10	15	20	5	5	5
**Theoretical Final concentration (mM)[a]**	0.2	0.2	0.2	0.2	0.2	0.2	0.2	0.2	0.4	0.5	0.6
**Ratio HAS/CYS**	10:5	1:1	1:2	1:3	1:5	1:1	1:1	1:1	1:1	1:1	1:1
**E.E.%[b]**	20.3	51.0	23.2	ND[d]	ND[d]	ND[d]	ND[d]	ND[d]	ND[d]	ND[d]	ND[d]
**E.E.%[c]**	ND[d]	ND[d]	ND[d]	ND[d]	ND[d]	ND[d]	ND[d]	ND[d]	ND[d]	ND[d]	ND[d]

[a]Calculated on the basis of the total amount of compound dissolved in the organic solvent; [b]at 37 °C; [c] at 45°C. [d]ND: compound not detected after filtration 0.2 μm syringe filter.

6) *Entrapment Efficacy (EE%)*

The percentage of entrapment efficacy was calculated with the following equation:

$$EE\% = \frac{Fi}{Ft} \times 100$$

where, *Fi* is the concentration of compound determined in the nano-preparation (evaluated by HPLC-UV-MS, after disruption of nanoparticles) and *Ft* is the theoretical final concentration (Calculated on the basis of the total amount of compound dissolved in the organic solvent).

7) *Liposome-drug nanoparticles preparations*

In order to define the best experimental conditions to obtain liposomes, different experiments were performed, varying several parameters (lipid ratio, use of sonication, drug concentration). Table S2 shows some representative experiments regarding our best liposomal candidate **LP-Si306**. The hydration volume was kept fixed at a value of 3 mL.

Table S2.							
**Batch. No.**	**(1) LP-Si306**	**(2) LP-Si306**	**(3) LP-Si 306**	**(4) LP-Si 306**	**(5) LP-Si 306**	**(6) LP-Si 306**	**(7) LP-Si 306**
**Lipids Molar Ratio**	6:3:1	6:3:1	20:10:1	20:10:1	20:10:1	20:10:1	20:10:1
**DPPC (mg)**	1.5	6.2	5.2	10.4	20.1	20.1	20.1
**Cholesterol (mg)**	0.4	1.6	1.3	2.7	5.5	5.5	5.5
**MPEG2000-DPPE (mg)**	1	4.0	1	2	4	4	4
**Theoretical Final concentration (mM)[a]**	0.1	0.1	0.1	0.1	0.4	0.5	0.6
**E.E.%[b]**	-	49.5	-	-	-	75.5	-
**E.E.%[c]**	27.1	52.3	79.4	98.7	98.2	99.1	59.6

[a]Calculated on the basis of the total amount of compound dissolved in the organic solvent. [b]with sonication at 40 °C. [c]with sonication at r.t.

8) Z-stack projection of SH-SY5Y cells incubated with fluorescent liposomes

Upper panel, Z-stack projection of SH-SY5Y cells incubated with fluorescent liposomes. Liposomes are visualized in green, cellular membrane in red and nuclei in blue. In the lower panel, dashed lines highlightes a liposome (green dot) and orthogonal projection of "X" and "Y" axis are shown. White arrows indicate the highlighted green dot inside the cell.

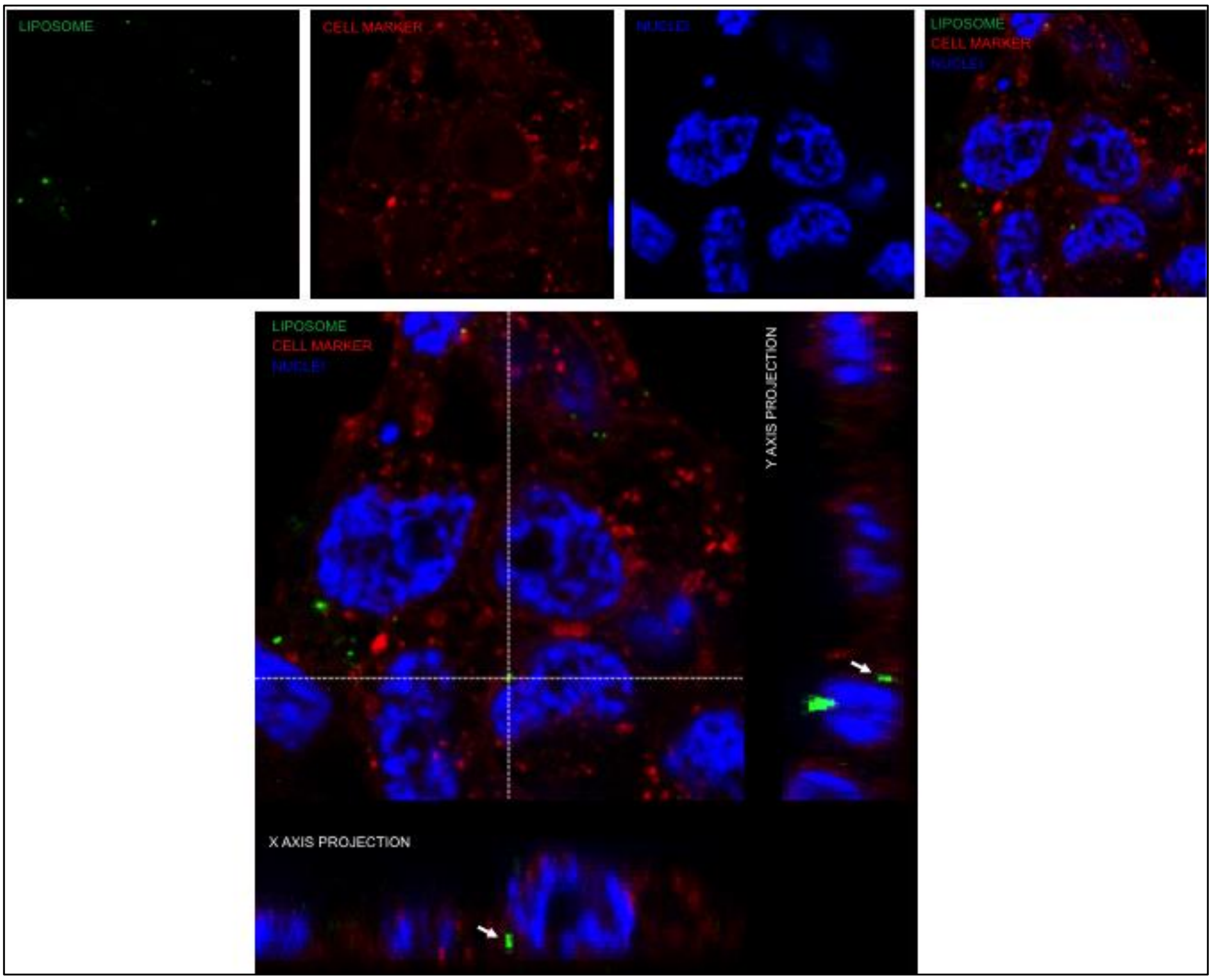

Figure S2. Z-stack projection of SH-SY5Y cells incubated with fluorescent liposomes
